# An unusual site for hydatid cyst on ovary misdiagnosed as an ovarian cyst: a case report

**DOI:** 10.1097/MS9.0000000000001004

**Published:** 2023-06-20

**Authors:** Hassan Bdeiwi, Hala Sultan, Zaher Mezketli, Tala jouma Al-hejazi, Mazen Trissi, Kinda Kellawi, Roula Zayat, AbdulMehdi Al-Hammod

**Affiliations:** a Faculty of Medicine, University of Aleppo; Departments of b General Surgery; c Obstetrics and Gynecology, Faculty of Medicine, University of Aleppo, Aleppo University Hospital, Aleppo, Syria

**Keywords:** case report, echinococcus, hydatid cyst, ovarian cyst, ovary

## Abstract

A hydatid cyst is a parasitic infection that can occur in multiple organs of the body, mostly in the liver. The ovary is one of the very rare locations for those cysts.

**Case presentation:**

The authors report a case of a 43-year-old woman with a primary hydatid cyst, the patient presented with left lower quadrant abdominal pain for 2 months. Ultrasound of the abdomen showed evidence of a multivesicular, fluid-containing cystic lesion in the left adnexa. The mass was excised and a hysterectomy with total left salpingo-oophorectomy was performed. Histopathology confirmed it to be a hydatid cyst.

**Clinical discussion:**

The clinical presentation of an ovarian hydatid cyst can differ, ranging from asymptomatic for years to dull pain if it compresses on the neighbouring organs or tissues, it may even cause a systemic immunological reaction if it ruptures.

**Conclusion:**

Cyst excision when possible is the best treatment, percutaneous sterilization techniques, and drug therapy may also be applied in certain cases.

## Introduction

Highlights

- Ovaries are rare places for hydatid cysts, yet a hydatid cyst should be among the differential diagnosis for a fluid-filled cyst on the ovary, especially in endemic areas.
- Hydatid cysts can mimic an ovarian cyst or a tumour and thus can lead to bad complications during excision in case it ruptures or in the case of a wrong diagnosis.
- Radical excision when possible can lead to great results and can prevent the possibility of rupturing which can lead to a deadly allergic reaction and can also decrease the recurrence possibility.

This article has been reported in line with the SCARE criteria^1^.

Echinococcosis is a zoonosis, that mammals form its intermediate host, humans can be accidental hosts as they do not play a role in the natural cycle. Dogs are the main definitive host. Hydatid cyst disease occurs through infection with the larval stages of the taeniid cestodes of the genus Echinococcus or through ingestion of free eggs that are found in food (unwashed vegetables), water, and infected soi. Those eggs are passed in the faeces of infected dogs and then ingested by humans. The larva then penetrates the intestinal wall and migrates via the bloodstream to all parts of the body^2^.

The liver is the most common location of hydatid cysts, and the second most common organ is the lungs^3^.

About 10% of the cysts can appear in unusual body sites. The ovary is one of the very rare locations for the cysts that causes ovarian hydatid disease (HD). It is morphologically similar to other locations and can occur primarily or secondary. Usually, the secondary is more common and is associated with liver or lung echinococcosis, or in the context of multiorgan hydatid cysts^3^.

Ovarian HD can stay asymptomatic for years but it can become symptomatic when a cyst compresses on the neighbouring organs or tissues, and the most common presentation is abdominal or pelvic pain^3^.

Imaging has an important role in the diagnosis and staging of the disease, imaging techniques include ultrasound which is one of the most common imaging modalities and can be used for the following process after treatment also, besides ultrasound we have computed tomography and MRI that can be useful when ultrasound does not show the cysts clearly or surgical treatment is required^4^.

Aside from imaging diagnosis, different serological tests have been introduced to diagnose hydatid cysts^4^.

In this paper, we report a rare case of a woman with an ovarian hydatid cyst, that was diagnosed and treated successfully in the obst&gyn hospital at the University of Aleppo.

## Case presentation

A 43-year-old woman presented to the hospital clinic with left lower quadrant abdominal pain increasing during menstruation for the last 2 months. There were no associated symptoms. She is a smoker but non-alcoholic, she does not take any medication, and her familial history has nothing important.

**Figure 1:**
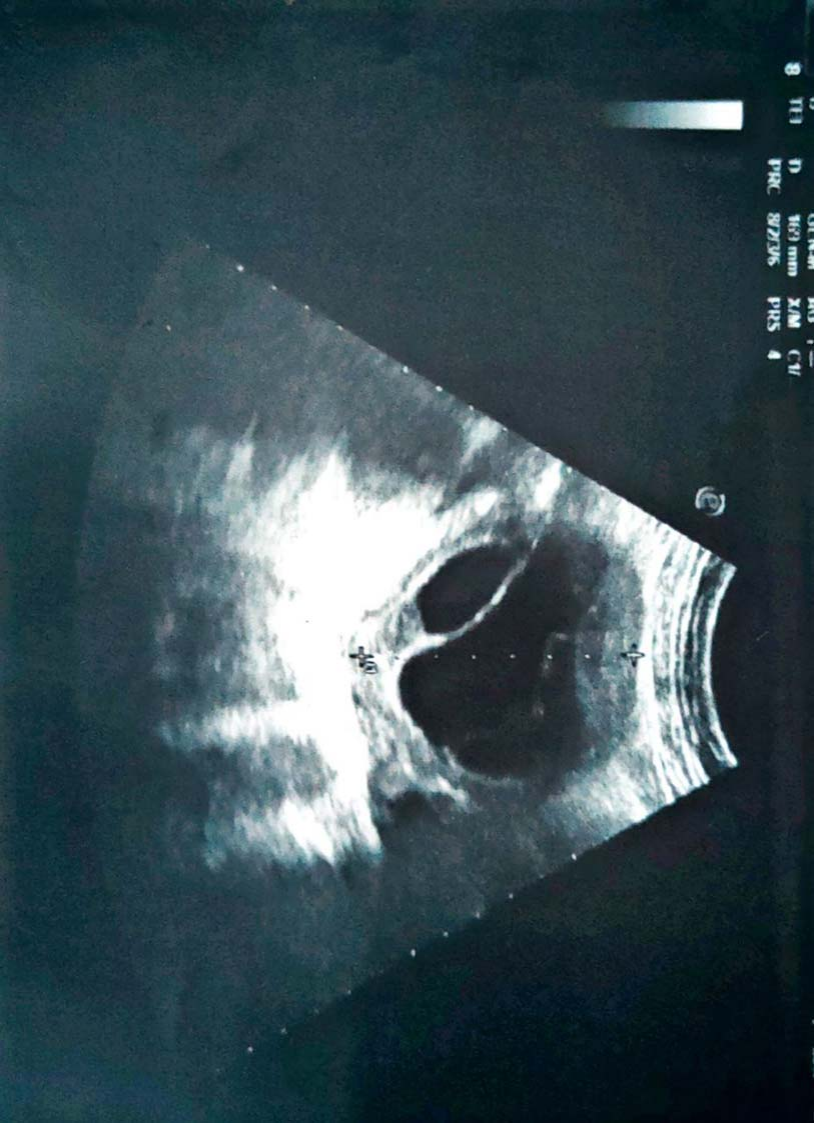
An ultrasound showing a well circumscribed, multivesicular, fluid-containing cyst.

Her past menstrual history was normal, she is gravida 8 and para7. No significant weight loss was reported lately. There has a history of a lipoma mass removal in the right flank. During the examination, the patient was not pale or jaundiced. No palpable organs or masses were detected. Laboratory investigations revealed normal liver and kidney functions with normal serum CA-125 and CEA. Ultrasound of the abdomen showed normal size and thickness of the uterus, and evidence of a multivesicular, fluid-containing cystic lesion in the left adnexa (9.5×8.5×4 cm approximately) while the right adnexa, liver, spleen, and kidneys were all normal (Fig. 1).

Taking these findings into consideration and after asking for the patient consent the operation was scheduled 2 days later.

Before the surgery routine lab tests were done, including CBCwbc: 9800 rbc: 3.9 hgb: 11.1 plt: 210*1000 CA15: 10.1 CEA: 1.84, urinalysis, and coagulation studies (PT/PTT), which all were normal.

**Figure 2:**
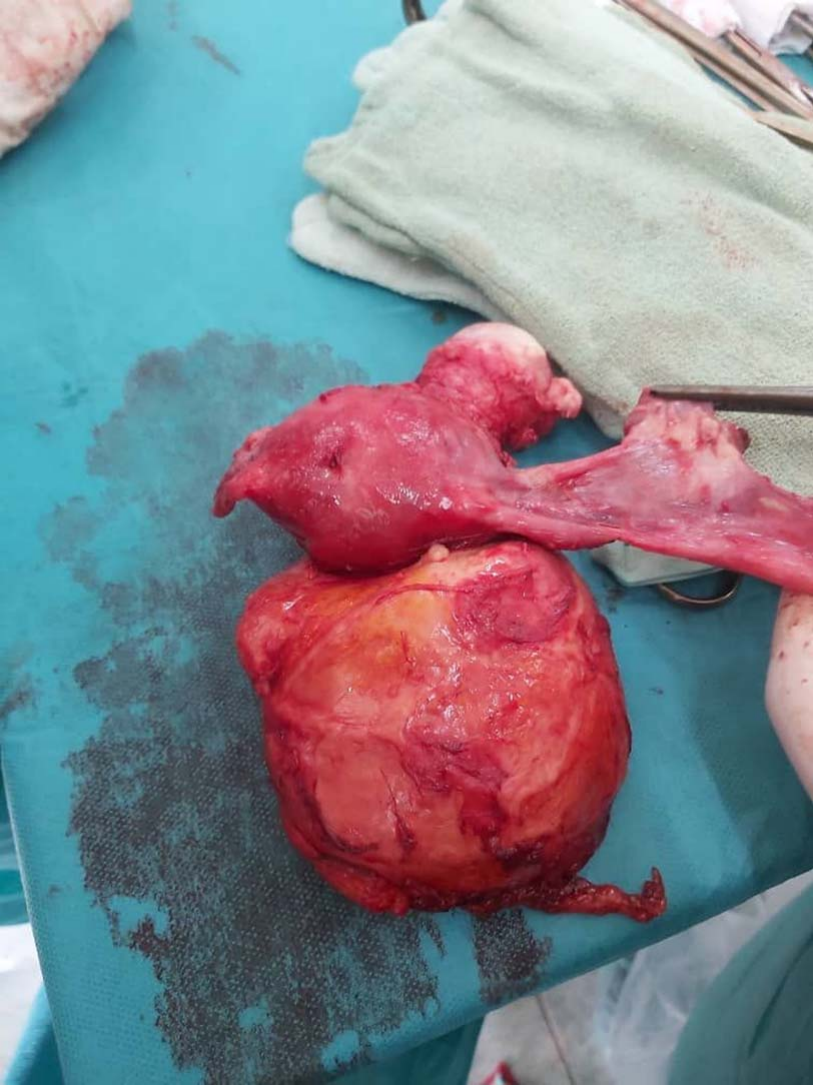
A gross image showing the size of the cyst comparing to the uterus after the operation.

**Figure 3:**
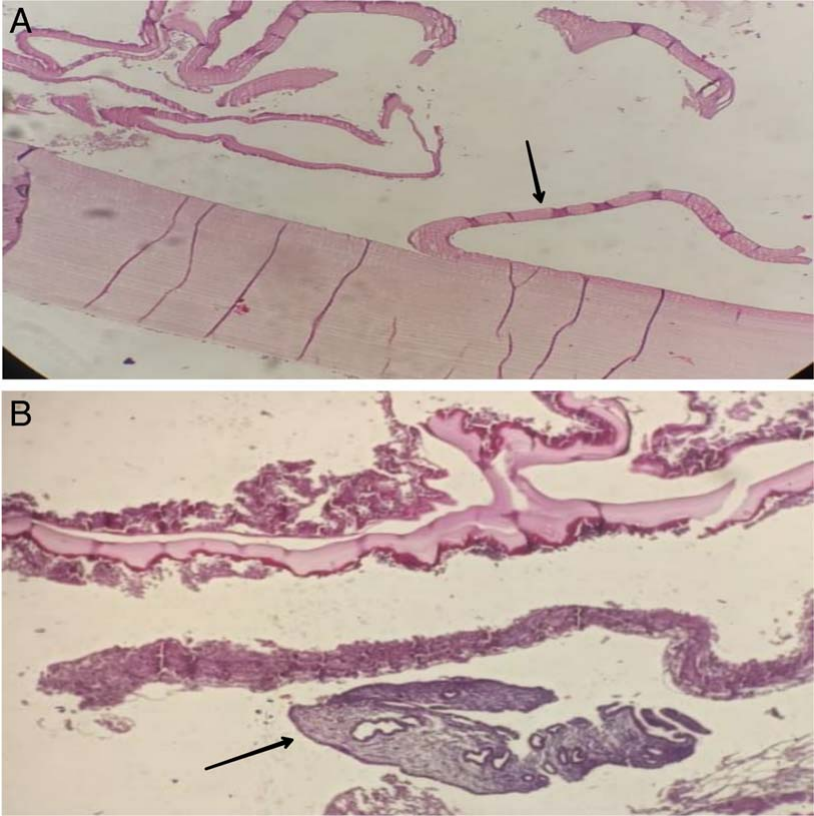
Histologic findings: (A) A hydatid cyst wall is seen, which is composed of an acellular laminated membrane (black arrow). (B) up the wall of the cyst appears, the black arrow refers to an exfoliated part of the endometrium that was in secretory phase.

During the surgical operation, the mass did not show the properties of a normal cyst. So, a hysterectomy with total left salpingo-oophorectomy was performed (Fig. 2). The histopathology reported it to be a cyst filled with yellowish fluid grossly and confirmed it to be a hydatid cyst microscopically (Fig. 3). The patient was admitted for 3 days after surgery and Albendazole was prescribed for 4 weeks. The patient was followed up in the preceding 6 months using abdominal ultrasound with no evidence of recurrence and the patient’s general condition has improved.

## Discussion

Echinococcosis is a zoonotic disease caused by tapeworms. It is divided into two forms: cystic echinococcosis, which is caused by Echinococcus granulosus sensu lato, and alveolar echinococcosis, which is caused by Echinococcus multilocularis^5^.

The disease is prevalent in certain areas, including western China, Central Asia, South America, Mediterranean countries, and eastern Africa^5^. The main risk factors for contracting the disease are contact with infected dogs and raising livestock^5^.

HD is caused by the larval stage of Echinococcus granulosus, and it can develop in almost any part of the body. The liver is the most common location for hydatid cysts, accounting for 75% of cases. The lungs are the second most common site, accounting for 15% of cases. The remaining 10% of cases occur in other areas of the body, such as the brain, cavernous sinus, submandibular gland, thyroid gland, heart, pleura, chest wall, retrocrural tissue, kidney, spleen, pancreas, peritoneal cavity, inguinal canal, breast, bone, and in some cases, the ovaries^6^. The clinical presentation for hydatid cysts can differ a lot ranging from asymptomatic for years to inducing systemic immunological reactions if it ruptures^7^. The onset of symptoms is related to the cyst’s size, position, organ, and correlation with adjacent tissues^7^.

As mentioned in our case, an ovarian hydatid cyst may mimic an ovarian tumour, which can lead to unnecessary procedures. Therefore, a precise diagnostic method is crucial. The gold standard method is abdominal ultrasonography, which has a sensitivity of 93%. Computed tomography may also be used. Other available diagnostic methods include serological tests, such as IgG against the parasite antigen with ELISA (sensitivity 95%) and the indirect hemagglutination test^8^.

Treatment options for hydatid cysts include surgery, which is the first choice according to the WHO, even though it does not prevent recurrence^9^.

However, chemotherapy with benzimidazole compounds (such as albendazole or mebendazole) and the recently developed PAIR procedure (puncture-aspiration-injection-re-aspiration), which destroys the cyst’s germinal layer, offer additional treatment options for CE cases. It is worth mentioning that chemotherapy is not a satisfactory cure, as only about 30% of patients can expect a cure, and 30–50% can expect improvement after 12 months of follow-up^9^.

In some cases, it may be reasonable to monitor the cyst rather than immediately pursuing treatment. This is because a proportion of cysts can calcify over time and become completely inactive^7^.

## Conclusion

Hydatid cyst should be considered among the differential diagnosis of a well-circumscribed ovarian cyst on echography, especially in endemic areas. It can be primary or secondary and can be asymptomatic for years. It is usually misdiagnosed as an ovarian cyst or an ovarian tumour and it can be well treated through radical excision and drug therapy. It should be followed to diagnose any recurrence. Careful excision is a must especially when an expected ovarian cyst does not show the properties of a normal cyst during surgery, as the rapture of the cyst can cause a dangerous allergic reaction.

## Ethics approval statement

Not applicable.

## Consent

Written informed consent was obtained from the patient for the publication of this case report and accompanying images. A copy of the written consent is available for review by the Editor-in-Chief of this journal on request.

## Source of funding

The authors declare no source of funding for this manuscript from any organization nor any institution.

## Author contribution

H.S., H.B., Z.M. and T.J.A.-H. wrote the manuscript. M.T., K.K. and R.Z.did the surgical operation and reviewed the manuscript. A.M.A.-H. reviewed and supervised the manuscript.

## Conflicts of interest disclosure

The authors want to declare no conflict of interest.

## Research registration unique identifying number (UIN)

None.

## Guarantor

Kinda Kellawi.

## Data availability statement

All data underlying the results are available as part of the article and no additional source data are required.

## Provenance and peer review

Not commissioned, externally peer-reviewed.
